# Dietary patterns in obese pregnant women; influence of a behavioral intervention of diet and physical activity in the UPBEAT randomized controlled trial

**DOI:** 10.1186/s12966-016-0450-2

**Published:** 2016-11-29

**Authors:** Angela C. Flynn, Paul T. Seed, Nashita Patel, Suzanne Barr, Ruth Bell, Annette L. Briley, Keith M. Godfrey, Scott M. Nelson, Eugene Oteng-Ntim, Sian M. Robinson, Thomas A. Sanders, Naveed Sattar, Jane Wardle, Lucilla Poston, Louise M. Goff, on behalf of the UPBEAT consortium

**Affiliations:** 1 Division of Women’s Health, King’s College London, 10th Floor North Wing, St. Thomas’ Hospital, London, UK; 2 Division of Diabetes and Nutritional Sciences, King’s College London, Franklin-Wilkins Building, 150 Stamford Street, London, UK; 3 Department of Cardiovascular Medicine, Imperial College London, London, UK; 4 Institute of Health & Society Newcastle University, UK Baddiley-Clark Building Richardson Road, Newcastle upon Tyne, UK; 5 MRC Lifecourse Epidemiology Unit and NIHR Southampton Biomedical Research Centre, University of Southampton and University Hospital Southampton NHS Foundation Trust, Mailpoint 95, Southampton, UK; 6 School of Medicine, University of Glasgow, New Lister Building, Glasgow Royal Infirmary, Glasgow, UK; 7 Guys and St Thomas’ NHS Foundation Trust, Westminster Bridge Road, London, UK; 8 Institute of Cardiovascular and Medical Sciences, RC214 Level C2, British Heart Foundation Glasgow Cardiovascular Research Centre, University of Glasgow, Glasgow, UK; 9 Health Behaviour Research Centre, Institute of Epidemiology and Health, University College London, 1-19 Torrington Place, London, UK

**Keywords:** Dietary patterns, Obesity, Pregnancy, Gestational diabetes

## Abstract

**Background:**

Understanding dietary patterns in obese pregnant women will inform future intervention strategies to improve pregnancy outcomes and the health of the child. The aim of this study was to investigate the effect of a behavioral intervention of diet and physical activity advice on dietary patterns in obese pregnant woman participating in the UPBEAT study, and to explore associations of dietary patterns with pregnancy outcomes.

**Methods:**

In the UPBEAT randomized controlled trial, pregnant obese women from eight UK multi-ethnic, inner-city populations were randomly assigned to receive a diet/physical activity intervention or standard antenatal care. The dietary intervention aimed to reduce glycemic load and saturated fat intake. Diet was assessed using a food frequency questionnaire (FFQ) at baseline (15^+0^-18^+6^ weeks’ gestation), post intervention (27^+0^-28^+6^ weeks) and in late pregnancy (34^+0^-36^+0^ weeks). Dietary patterns were characterized using factor analysis of the baseline FFQ data, and changes compared in the control and intervention arms. Patterns were related to pregnancy outcomes in the combined control/intervention cohort (*n* = 1023).

**Results:**

Four distinct baseline dietary patterns were defined; Fruit and vegetables, African/Caribbean, Processed, and Snacks, which were differently associated with social and demographic factors. The UPBEAT intervention significantly reduced the Processed (−0.14; 95% CI −0.19, −0.08, *P* <0.0001) and Snacks (−0.24; 95% CI −0.31, −0.17, *P* <0.0001) pattern scores. In the adjusted model, baseline scores for the African/Caribbean (quartile 4 compared with quartile 1: *OR* = 2.46; 95% CI 1.41, 4.30) and Processed (quartile 4 compared with quartile 1: *OR* = 2.05; 95% CI 1.23, 3.41) patterns in the entire cohort were associated with increased risk of gestational diabetes.

**Conclusions:**

In a diverse cohort of obese pregnant women an intensive dietary intervention improved Processed and Snack dietary pattern scores. African/Caribbean and Processed patterns were associated with an increased risk of gestational diabetes, and provide potential targets for future interventions.

**Trial registration:**

Current controlled trials; ISRCTN89971375

**Electronic supplementary material:**

The online version of this article (doi:10.1186/s12966-016-0450-2) contains supplementary material, which is available to authorized users.

## Background

Obesity represents a significant and increasing global public health problem [1]. In pregnancy, because of associations with increased risk of gestational diabetes (GDM) [2] and many other maternal and fetal complications [3], maternal obesity has become one of the most challenging health care problems in 21^st^ century obstetrics.

The pivotal role of maternal nutrition in pregnancy is well established, with several studies demonstrating the importance of nutritional intake and status during pregnancy, both for short-term pregnancy outcomes [4–6], and long-term health of the offspring [5, 7–13]. A concerted effort has therefore been made towards identifying interventions to improve the nutrition of obese pregnant women and prevent adverse pregnancy outcomes. The predominant focus has been on randomized controlled trials (RCTs) of dietary interventions initiated during pregnancy, with or without physical activity with the intention of reducing gestational weight gain or improving glucose tolerance. However, systematic review of these studies, and large scale RCTs adequately powered for clinical outcomes, including a recent report from our group have shown that these interventions during pregnancy are ineffective in prevention of GDM, large for gestational age (LGA) infants, or other adverse outcomes [14–18]. The failure of these RCTs has shifted the focus to targeted interventions for those individuals identified at greatest risk.

Nutritional assessment in dietary intervention studies has traditionally focused on evaluating the effects of individual nutrients or foods on health outcomes. However, the limitations of this approach are becoming more evident as the role of isolated nutrients or foods is increasingly difficult to ascertain [19]. Assessing dietary patterns provides an alternative approach by examining combinations and clustering of foods and nutrients, thus representing the overall diet [20]. Better understanding of dietary patterns in obese pregnant women may provide a means to identify unhealthy dietary habits and their associations with pregnancy outcomes, thus informing potential specific targeted interventions.

In this study, we aimed to investigate dietary patterns in obese pregnant women who participated in the UK Pregnancies Better Eating and Activity Trial (UPBEAT) RCT, to assess the effects of the intervention and to examine associations between the baseline maternal dietary patterns and adverse pregnancy outcomes with the aim of informing new targets for intervention.

## Methods

### Study design and setting

A detailed protocol and the trial outcome have been published [17, 21]. In brief, UPBEAT was a multicenter randomized controlled trial based in eight UK inner city NHS Trust Hospitals [Bradford, Glasgow, London (three centers), Manchester, Newcastle, and Sunderland]. Ethical approval was granted by the NHS Research Ethics Committee (UK IRAS, Integrated Research Application System; reference 09/H0802/5). All participants provided written informed consent.

### Patient selection

Participants with a body mass index (BMI) ≥30 kg/m^2^ and a singleton pregnancy between 15^+0^- 18^+6^ weeks’ gestation were eligible for enrolment. Participants of <15^+0^ or >18^+6^ weeks’ gestation, individuals with underlying disease or those unable or unwilling to give informed consent were ineligible.

### Blinding and random assignment

Participants were allocated to the intervention and standard antenatal care or to standard antenatal care alone using a computer generated randomization procedure via a password protected website (MedSciNet™). Randomization was minimized according to ethnicity (Black, White, Asian, other), parity (nulliparous versus multiparous), BMI (30.0-34.9, 35.0-39.9, ≥40 kg/m^2^), age (≤24, 25–29, 30–34, ≥ 35 years) and center. Due to the nature of the intervention, participants and staff were aware of the allocation.

### Intervention

Participants randomized to the intervention group participated in a behavioral intervention of diet and physical activity advice, which was delivered by health trainers. Within a week of randomization, the participants attended a one-to-one session with the health trainer, followed by eight consecutive weekly individual or group sessions. The dietary intervention did not restrict energy intake but aimed to promote a healthier pattern of eating, focusing principally on achieving two dietary goals: a reduction in dietary glycemic load (GL) (50 unit reduction) and a reduction in saturated fat intake (<10% of energy). In order to decrease GL, dietary advice included exchange of starchy foods with medium/high glycemic index (GI) for those with a lower GI and restricting the consumption of sugar-sweetened beverages including fruit juice. To reduce saturated fat intake participants were encouraged to use low fat dairy products and replace fatty meats and meat products with lean meat or fish. UPBEAT targeted dietary advice is detailed in Additional file 1: Table S1. In total, there were eight key dietary changes and each session had a specific goal. The participants received a handbook with detailed guidance and tips on making the changes, along with recipe ideas and more general information on eating while pregnant and a logbook to record their dietary goals. Each session addressed approaches to achieving SMART (Specific, Measurable, Achievable, Relevant, Time Specific) goals. Additionally, participants were advised on self-monitoring, identification and problem solving of barriers to behaviour change, enlisting social support and providing opportunities for social comparison.

Physical activity advice focused on incrementally increasing walking and being more active in daily life. Walking at a moderate intensity was encouraged and pedometers were provided for motivation and self-monitoring purposes.

### Standard care

All participants attended antenatal appointments according to local health care provision at their study centres. For those randomized to standard care, no additional information was provided.

Participants from both arms of the trial had an oral glucose tolerance test (75 g glucose load) at 27^+0^ to 28^+6^ weeks gestation for GDM diagnosis. GDM diagnostic criteria were as recommended by the International Association of Diabetes and Pregnancy Study Groups (IADPSG) [22]. Participants were referred for GDM management according to local guidelines in each centre.

### Assessment of dietary intake

Dietary assessment was performed by study specific research midwives at baseline (15^+0^-18^+6^ weeks gestation), post intervention (27^+0^-28^+6^ weeks gestation) and in late pregnancy (34^+0^-36^+0^ weeks gestation), beyond the active intervention phase. Diet was assessed in all participants using a semi-quantitative food frequency questionnaire (FFQ) adapted from the UK arm of the European Prospective Investigation into Cancer Study (EPIC) [23]. The FFQ was a shortened version (50 items) of the EPIC questionnaire and focused primarily on assessing intake of food groups relevant to the UPBEAT intervention. Questions relating to sources of carbohydrate were detailed to distinguish low GI (e.g. multigrain and granary breads, porridge, pasta, basmati rice, new potatoes) from high GI varieties (e.g. white bread, refined breakfast cereals, easy cook rice, old potatoes) and questions relating to dietary fat distinguished high saturated fat sources (e.g. full fat dairy products, fatty meats and meat products) from low saturated fat varieties (e.g. low fat dairy products, lean meat, chicken and fish). Accompanying the list was a multiple response grid in which the participants estimated frequency of consumption of foods eaten over the preceding month, ranging from never or less than once a month to 6 or more times per day. Alcohol consumption was not assessed in the FFQ; intake was recorded separately with 95% of participants reporting alcohol abstinence at the first study visit. Using Pearson correlation coefficient, the FFQ was compared to 24 h recalls collected from the pilot study participants [24] and showed good agreement for fat (*r* = 0.28, *P* = 0.017), saturated fat (*r* = 0.26, *P* = 0.020), protein (*r* = 0.25, *P* =0.028) and sugar (*r* = 0.32, *P* = 0.004).

A program was developed in collaboration with the trial database team to transform data from the FFQs into nutrient intakes. WISP 3.0 (Tinuviel Software) dietary analysis software was used to calculate nutritional composition and GL/100 g for each line on the FFQ. WISP calculates GL based on the GI and carbohydrate content of each food and using the following formula: GI of each food x CHO amount/100 using previously published GI values [25]. Where GI values were missing or required updating, additional UK [26] and more recent published values [27] were inputted. Average portion sizes were obtained from national references [28, 29] and conversion factors were applied to convert frequency of consumption to daily nutrient intakes [30].

### Extraction of dietary patterns

Factor analysis with orthogonal rotation was performed on the baseline dietary data to derive dietary patterns using the participants’ intake of each of the 50 food groups listed on the FFQ (the food groups considered for identification of the dietary patterns are described in Additional file 2: Table S2). Questionnaires with missing data were excluded from the analysis. The number of factors that best represented the data was chosen on the basis of the scree plot of eigenvalues and the interpretability of factor loadings. Following orthogonal rotation, food groups with a factor loading of ≥ ± 0.25 were considered to have a strong association with that factor, and a scoring system was derived using standard methods. To assess changes in dietary patterns following the UPBEAT intervention, this scoring system, derived from baseline data at 16–18 weeks only, was applied to diet at 27–28 weeks and 34–36 weeks, and up to three sets of applied dietary pattern scores were calculated for each participant [31].

### Statistical analysis

Normality of dietary patterns was investigated using distributional plots. To test for the effect of the intervention on dietary pattern scores between the control and intervention groups at 28 and 36 weeks, analysis of covariance (ANCOVA) was used, adjusted for trial entry measurements. As preliminary analysis demonstrated no difference in dietary pattern scores between the control and intervention groups at baseline (data not shown), the data from the groups was pooled to investigate the association between baseline dietary patterns and social and demographic factors and to examine the effect of baseline dietary patterns on pregnancy outcomes. Multiple linear regression was carried out to examine the association between dietary pattern scores and social and demographic factors, adjusted for age, ethnicity, education, living in a deprived area and parity. Adjusted regression coefficients and 95% confidence intervals (95% CI) are presented..Multiple logistic regression models were constructed to examine the association between pregnancy outcomes and dietary patterns for one standard deviation difference in the factor score. The specific outcomes examined included GDM defined by IADPSG criteria, LGA infant (*≥*90^th^ population birthweight centile calculated with WHO centiles), small for gestational age (SGA) infant (≤10^th^ population birthweight centile), macrosomia (birthweight ≥4 kg) and pre-eclampsia (defined as systolic blood pressure ≥140 mm Hg, diastolic blood pressure ≥90 mm Hg, or both, on at least two occasions 4 h apart, with proteinuria ≥300 mg/24 h or spot urine protein:creatinine ratio ≥30 mg/mmol creatinine, or urine dipstick protein ≥2+). The main analysis was carried out using factor scores (mean 0, SD 1); but key results were repeated as comparisons of the lowest and highest quarters of the distribution. The models were adjusted for age, parity, ethnicity, BMI, living in a deprived area and treatment allocation. Statistical analysis was carried out using Stata version 13 (StataCorp, College Station, Texas).

## Results

UPBEAT was conducted from March 2009 to May 2014 with 1555 women randomized to intervention or standard care groups [17]. A total of 1023 participants had complete food group data to extract dietary patterns [excluded due to incomplete questionnaires (*n* = 349) and pilot study participants assessed by 24 h recalls (*n* = 183)]. The demographic characteristics of these women with complete food group data are shown in Table 1. The mean age (SD) of the participants was 30.5 (SD 5.5) years and the mean BMI was 36.2 (SD 4.7) kg/m^2^. The majority of women were of White (64%) ethnicity and the remainder from Black (23%), Asian (8%) and other ethnic minority groups. More than half of participants had at least one child (57%) and 43% lived in a deprived area according to the Index of Multiple Deprivation. To investigate the effect of excluding participants, we compared those with missing data with the whole sample (*n* = 1555). The only difference in the baseline characteristics was for ethnicity: the subsample had less representation from Black participants (data not shown).

**Table 1:** Maternal characteristics of the UPBEAT participants with complete food group data

	Whole group	Control	Intervention
	(*n* = 1023)	(*n* = 504)	(*n* = 519)
Age (years)	30.5 (5.5)	30.5 (5.7)	30.4 (5.3)
Ethnicity			
White	652 (64%)	322 (64%)	330 (64%)
Black	236 (23%)	115 (23%)	121 (23%)
Asian	79 (8%)	40 (8%)	39 (8%)
Other	56 (5%)	27 (5%)	29 (6%)
BMI (kg/m^2^)^a^	36.2(4.7)	36.2 (4.6)	36.2 (4.9)
Parity			
Nulliparous	440 (43%)	220 (44%)	220 (42%)
Multiparous	583 (57%)	284 (56%)	299 (58%)
Education^b^			
None/GCSE	203 (20%)	103 (20%)	100 (19%)
A level	165 (16%)	82 (16%)	83 (16%)
Degree/higher degree	408 (40%)	197 (39%)	211 (41%)
Vocational qualification	247 (24%)	122 (24%)	125 (24%)
Index of multiple deprivation^c^			
1 (least deprived)	43 (4%)	26 (5%)	17 (3%)
2	74 (7%)	32 (6%)	42 (8%)
3	124 (12%)	53 (11%)	71 (14%)
4	345 (34%)	192 (38%)	153 (30%)
5 (most deprived)	436 (43%)	201 (40%)	235 (45%)

Results shown are mean (SD) or n (%)

^a^ *BMI* body mass index

^b^ *GCSE* General Certificate of Secondary Education, *A-level* General Certificate of Education Advanced Level

^c^Scores were calculated for the region of residence, by fifths of the population. UK-wide scores were developed from English and Scottish data relating to employment and income domains

### Dietary patterns identified

**Fig. 1:**
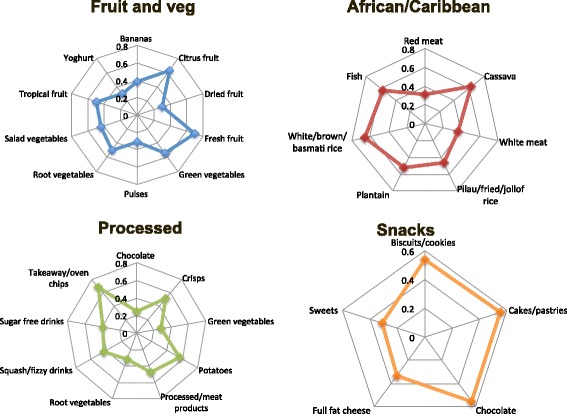
Factor loadings ≥ 0.25 for each identified dietary pattern

Four factors were identified using factor analysis. Figure 1 shows spiderplots of factor loadings ≥ ± 0.25 for the four patterns. The full list of factor loadings is shown in Additional file 3: Table S3. The first factor was characterized by high intakes of bananas, citrus fruit, dried fruit, fresh fruit, green vegetables, pulses, root vegetables, salad vegetables, tropical fruit and yoghurt. Factor 1 was termed the ‘Fruit and vegetables’ dietary pattern. The second factor was labeled the ‘African/Caribbean’ dietary pattern due to its high loadings on red meat, cassava, white meat, rice including pilau, fried or jollof rice, plantain and fish. The third factor derived was characterized by intakes of chocolate, crisps, green vegetables, potatoes, processed meat and meat products, root vegetables, squash and fizzy drinks, sugar free squash and fizzy drinks and chips. This factor was termed the ‘Processed’ dietary pattern. The fourth factor was labeled the ‘Snacks’ dietary pattern due to high loadings on biscuits, cookies, cakes, pastries, chocolate, full fat cheese and sweets.

### Effect of the UPBEAT intervention on dietary pattern scores

The effect of the UPBEAT intervention on dietary pattern scores is shown in Table 2. Following the intervention (28 weeks), there were significant reductions in the Processed (−0.14; 95% CI −0.19, −0.08, *P* <0.0001) and Snacks (−0.24; 95% CI −0.31, −0.17, *P* <0.0001) dietary pattern scores in the intervention group which were maintained in late gestation (36 weeks). There was no change between the groups in scores for the Fruit and vegetables and African/Caribbean dietary patterns at 28 and 36 weeks gestation.

**Table 2:** Dietary pattern scores at baseline (15^+0^-18^+6^ weeks’ gestation), following the UPBEAT intervention (27^+0^-28^+6^ weeks’ gestation) and in late gestation (34^+0^ to 36^+0^ weeks’ gestation)

Dietary pattern		Control^a^	Intervention^b^	Difference (95% CI)	*P*
Fruit and veg	Baseline	0.04 (0.99)	−0.04 (0.78)		
	28 weeks	−0.08 (0.79)	−0.03 (0.84)	0.07 (−0.02 to 0.17)	0.135
	36 weeks	−0.15 (0.82)	−0.09 (0.79)	0.07 (−0.04 to 0.17)	0.208
African/Caribbean	Baseline	−0.03 (0.76)	0.02 (0.97)		
	28 weeks	−0.06 (0.74)	−0.07 (0.60)	−0.03 (−0.10 to 0.04)	0.433
	36 weeks	−0.13 (0.54)	−0.11 (0.52)	0.00 (−0.06 to 0.07)	0.916
Processed	Baseline	0.02 (1.10)	−0.02 (0.56)		
	28 weeks	−0.04 (0.52)	−0.18 (0.49)	−0.14 (−0.19 to −0.08)	<0.0001
	36 weeks	−0.06 (0.57)	−0.16 (0.50)	−0.09 (−0.16 to −0.02)	0.011
Snacks	Baseline	−0.03 (0.75)	0.03 (0.91)		
	28 weeks	0.04 (0.69)	−0.18 (0.55)	−0.24 (−0.31 to −0.17)	<0.0001
	36 weeks	−0.05 (0.66)	−0.13 (0.69)	−0.11 (−0.20 to −0.02)	0.018

Results are reported as mean (SD)

^a^Control; *n* = 504 at baseline (15^+0^-18^+6^ weeks’ gestation), 436 following the UPBEAT intervention (27^+0^-28^+6^ weeks’ gestation) and 363 in late gestation (34^+0^ to 36^+0^ weeks’ gestation)

^b^Intervention; *n* = 519 at baseline (15^+0^-18^+6^ weeks’ gestation), 417 following the UPBEAT intervention (27^+0^-28^+6^ weeks’ gestation) and 325 in late gestation (34^+0^ to 36^+0^ weeks’ gestation)

### Variation with social and demographic factors

Dietary pattern scores were differently associated with social and demographic factors, which were robust to adjustment (Table 3). Participants with lower educational attainment had lower scores on the Fruit and vegetables pattern. The African/Caribbean pattern score was higher in ethnic minority groups compared to White participants and lower in nulliparous participants. Younger participants had higher scores on the Processed pattern and scores for this pattern were lower in ethnic minority groups and in nulliparous participants. Black participants and nulliparous participants had lower scores on the Snacks pattern.

**Table 3:** Adjusted linear regression coefficients (95% confidence interval) for baseline dietary pattern scores according to social and demographic factors^a^

	Fruit and veg	African/ Caribbean	Processed	Snacks
	Coefficient 95% CI	Coefficient 95% CI	Coefficient 95% CI	Coefficient 95% CI
Age				
< 20	−0.19 (−0.56, 0.18)	−0.1 (−0.24, 0.05)	0.28 (0.00, 0.56)	−0.04 (−0.31, 0.23)
21-25	−0.14 (−0.31, 0.03)	0 (−0.14, 0.15)	0.27 (0.12, 0.43)	−0.09 (−0.23, 0.06)
26-30	−0.14 (−0.29, 0.00)	0.05 (−0.07, 0.17)	0.15 (−0.03, 0.34)	−0.09 (−0.23, 0.04)
31-35	Reference			
36+	−0.03 (−0.17, 0.11)	−0.06 (−0.20, 0.09)	−0.02 (−0.11, 0.07)	−0.05 (−0.21, 0.12)
*P*	0.262	0.217	0.004	0.701
Ethnicity				
White	Reference			
Asian	0.23 (−0.01, 0.48)	0.28 (0.14, 0.42)	−0.24 (−0.40, −0.08)	−0.1 (−0.32, 0.12)
Black	0.08 (−0.06, 0.21)	1.01 (0.83, 1.19)	−0.34 (−0.46, −0.22)	−0.41 (−0.55, −0.28)
Other	0.06 (−0.19, 0.32)	0.24 (0.11, 0.38)	−0.11 (−0.34, 0.12)	−0.17 (−0.40, 0.06)
*P*	0.248	<0.0001	<0.0001	<0.0001
Education^b^				
Degree	Reference			
A level	−0.2 (−0.37, −0.03)	0.01 (−0.15, 0.16)	0.08 (−0.08, 0.23)	−0.06 (−0.19, 0.07)
None/GCSE	−0.22 (−0.39, −0.05)	0.04 (−0.15, 0.22)	0.18 (0.01, 0.35)	0 (−0.13, 0.14)
Vocational	−0.33 (−0.47, −0.19)	−0.06 (−0.17, 0.05)	0.11 (−0.06, 0.28)	−0.03 (−0.18, 0.13)
*P*	<0.0001	0.378	0.215	0.822
Deprivation^c^				
1 (least deprived)	0.05 (−0.15, 0.26)	−0.05 (−0.19, 0.09)	−0.11 (−0.31, 0.10)	−0.07 (−0.32, 0.18)
2	−0.08 (−0.34, 0.19)	−0.1 (−0.23, 0.04)	−0.09 (−0.22, 0.04)	−0.02 (−0.21, 0.18)
3	−0.03 (−0.22, 0.16)	−0.04 (−0.19, 0.10)	0.02 (−0.28, 0.33)	−0.19 (−0.33, −0.05)
4	0 (−0.12, 0.12)	−0.11 (−0.22, 0.00)	−0.12 (−0.20, −0.03)	−0.13 (−0.25, −0.02)
5 (most deprived)	Reference			
*P*	0.937	0.410	0.110	0.063
Parity				
Nulliparous	−0.01 (−0.13, 0.11)	−0.14 (−0.22, −0.06)	−0.16 (−0.31, −0.01)	−0.12 (−0.22, −0.01)
Multiparous	Reference			
*P*	0.855	0.001	0.039	0.027

^a^Dietary pattern score as the outcome variable and social and demographic factors (age, ethnicity, education, deprivation, parity) as exposure variables

^b^ *GCSE* General Certificate of Secondary Education, *A-level* General Certificate of Education Advanced Level

^c^Scores were calculated for the region of residence, by fifths of the population. UK-wide scores were developed from English and Scottish data relating to employment and income domains

### Association with pregnancy outcomes

The association of baseline dietary patterns with pregnancy outcomes is shown in Table 4. The baseline African/Caribbean and Processed dietary patterns were associated with an increased risk of developing GDM. Participants in the highest quartile of the African/Caribbean (*OR* = 2.46; 95% CI 1.41, 4.30) and Processed (*OR* = 2.05; 95% CI 1.23, 3.41) dietary patterns had a higher risk of developing GDM compared with participants in the lowest quartile. These associations were robust to adjustment for confounders. There was no association between the dietary patterns and delivering a LGA, SGA or macrosomic infant or with the development of pre-eclampsia.

**Table 4:** Association between baseline dietary pattern scores and risk of adverse pregnancy outcomes (odds ratio and 95% confidence intervals)^a^

Pregnancy outcomes	Incidence	Dietary pattern		Odds Ratio	95% CI
GDM (*n* = 857)	200 (23%)	Fruit and veg	Quartile 1	Reference	
			Quartile 2	1.13	(0.71, 1.81)
			Quartile 3	1.18	(0.73, 1.89)
			Quartile 4	1.03	(0.64, 1.68)
			*P*		0.891
		African/Caribbean	Quartile 1	Reference	
			Quartile 2	1.26	(0.79, 1.99)
			Quartile 3	1.13	(0.70, 1.82)
			Quartile 4	2.46	(1.41, 4.31)
			*P*		0.010
		Processed	Quartile 1	Reference	
			Quartile 2	1.71	(1.04, 2.82)
			Quartile 3	2.03	(1.23, 3.33)
			Quartile 4	2.05	(1.23, 3.41)
			*P*		0.022
		Snacks	Quartile 1	Reference	
			Quartile 2	0.94	(0.57, 1.54)
			Quartile 3	1.10	(0.67, 1.81)
			Quartile 4	1.24	(0.76, 2.01)
			*P*		0.666
LGA^b^(*n* = 995)	109 (11%)	Fruit and veg	Quartile 1	Reference	
			Quartile 2	1.39	(0.76, 2.53)
			Quartile 3	1.41	(0.77, 2.59)
			Quartile 4	1.70	(0.94, 3.06)
			*P*		0.377
		African/Caribbean	Quartile 1	Reference	
			Quartile 2	1.41	(0.79, 2.50)
			Quartile 3	1.52	(0.85, 2.71)
			Quartile 4	1.47	(0.73, 2.97)
			*P*		0.512
		Processed	Quartile 1	Reference	
			Quartile 2	0.93	(0.52, 1.67)
			Quartile 3	0.77	(0.42, 1.41)
			Quartile 4	0.85	(0.46, 1.55)
			*P*		0.844
		Snacks	Quartile 1	Reference	
			Quartile 2	1.10	(0.59, 2.07)
			Quartile 3	1.38	(0.74, 2.57)
			Quartile 4	1.14	(0.60, 2.15)
			*P*		0.749
SGA^c^(*n* = 995)	61 (6%)	Fruit and veg	Quartile 1	Reference	
			Quartile 2	0.38	(0.17, 0.83)
			Quartile 3	0.66	(0.33, 1.32)
			Quartile 4	0.48	(0.23, 1.03)
			*P*		0.073
		African/Caribbean	Quartile 1	Reference	
			Quartile 2	1.30	(0.62, 2.72)
			Quartile 3	0.46	(0.19, 1.16)
			Quartile 4	1.10	(0.46, 2.65)
			*P*		0.128
		Processed	Quartile 1	Reference	
			Quartile 2	1.82	(0.86, 3.86)
			Quartile 3	1.49	(0.66, 3.38)
			Quartile 4	1.48	(0.65, 3.40)
			*P*		0.479
		Snacks	Quartile 1	Reference	
			Quartile 2	0.93	(0.45, 1.90)
			Quartile 3	0.68	(0.30, 1.53)
			Quartile 4	0.76	(0.36, 1.63)
			*P*		0.773
Macrosomia^d^(*n* = 997)	133 (13%)	Fruit and veg	Quartile 1	Reference	
			Quartile 2	0.86	(0.49, 1.51)
			Quartile 3	1.33	(0.79, 2.27)
			Quartile 4	1.40	(0.83, 2.36)
			*P*		0.236
		African/Caribbean	Quartile 1	Reference	
			Quartile 2	1.45	(0.86, 2.45)
			Quartile 3	1.71	(1.01, 2.88)
			Quartile 4	0.98	(0.50, 1.94)
			*P*		0.114
		Processed	Quartile 1	Reference	
			Quartile 2	0.78	(0.45, 1.33)
			Quartile 3	0.70	(0.40, 1.21)
			Quartile 4	0.95	(0.55, 1.61)
			*P*		0.520
		Snacks	Quartile 1	Reference	
			Quartile 2	1.67	(0.92, 3.02)
			Quartile 3	1.69	(0.93, 3.09)
			Quartile 4	1.69	(0.93, 3.08)
			*P*		0.286
Pre-eclampsia (*n* = 984)	36 (4%)	Fruit and veg	Quartile 1	Reference	
			Quartile 2	0.88	(0.36, 2.13)
			Quartile 3	0.58	(0.22, 1.54)
			Quartile 4	0.61	(0.23, 1.66)
			*P*		0.641
		African/Caribbean	Quartile 1	Reference	
			Quartile 2	0.79	(0.35, 1.80)
			Quartile 3	0.42	(0.15, 1.14)
			Quartile 4	0.42	(0.12, 1.45)
			*P*		0.298
		Processed	Quartile 1	Reference	
			Quartile 2	0.86	(0.32, 2.33)
			Quartile 3	0.51	(0.16, 1.62)
			Quartile 4	1.40	(0.55, 3.54)
			*P*		0.291
		Snacks	Quartile 1	Reference	
			Quartile 2	1.14	(0.40, 3.23)
			Quartile 3	1.41	(0.51, 3.94)
			Quartile 4	1.22	(0.43, 3.46)
			*P*		0.924

Abbreviations; *GDM* gestational diabetes, *LGA* large for gestational age, *SGA* small for gestational age

^a^ Adjusted for age, parity, ethnicity, BMI, living in a deprived area and treatment allocation

^b^ Large for gestational age defined as >90^th^ WHO centile

^c^ Small for gestational age defined as <10^th^ WHO centile

^d^Macrosomia defined as birthweight >4 kg

## Discussion

In this study, we have identified four distinct dietary patterns in obese pregnant women, which differed relative to demographic and social factors. Two dietary patterns; African/Caribbean and Processed were associated with GDM at trial entry in adjusted analyses. The UPBEAT intervention reduced the Processed and Snacks dietary pattern scores; however, despite the Processed pattern being associated with GDM, the modest reduction was not clinically significant.

The present study confirms a preliminary report from the UPBEAT pilot study [32] and provides the first and most extensive analysis of dietary patterns in obese pregnant women. Two unhealthy dietary patterns were recognized at baseline: the Processed and Snacks patterns, which were characterized by foods and beverages high in sugar and/or fat, including saturated fat, and are in line with several studies that identified similar unhealthy dietary patterns in pregnant women with heterogeneous BMI [33–36]. Consistent with previous reports, these unhealthy patterns highlight the poor quality diets consumed by obese pregnant women [37–40].

Furthermore, two additional patterns were identified: the Fruit and vegetables pattern, consistent with dietary patterns described in several pregnant populations, was characterized by high intakes of fruit and vegetables [33, 36, 41, 42]; and the African/Caribbean pattern, which included rice, cassava and plantain, reflected the ethnic diversity of the UPBEAT participants, in which 23% of participants were of Black African or Caribbean ethnicity. A previous report of the diets of Black British adults has shown that, in both West African and Caribbean diets, rice dishes were the main source of energy. Furthermore, cassava, traditional red meat and fish stews were important contributors to energy in the diets of West African adults [43]. The African/Caribbean pattern is consistent with this assessment and recognizes the importance of traditional, cultural foods in the diets of ethnic minority groups in the UK.

In relation to the UPBEAT intervention, reductions in the Processed and Snacks scores were evident, which suggests that the trial participants were receptive to improving their diet. This is in agreement with the Australian LIMIT study, which applied a dietary index in pregnant women who were overweight or obese to derive dietary patterns and showed that women randomized to lifestyle advice demonstrated a significant improvement in the index score during pregnancy [44]. Furthermore, we found that these dietary pattern changes were sustained beyond the active intervention phase, which may be a reflection of the intensity of the intervention and the focus on behavioral theory. This is of some importance as it is well recognized that diet and/or physical activity changes at least in the non-pregnant population are difficult to maintain [45].

The intervention had no effect on the Fruit and vegetables or the African/Caribbean dietary patterns. The dietary habits of minority ethnic groups are affected by a wide variety of factors including income, socio-economic status, religious beliefs, food availability and food beliefs [46]. Whilst appreciating potential resistance to change, independently of ethnicity this African/Caribbean pattern was associated with increased risk of GDM and it remains a potential target for intervention.

We identified important associations between dietary patterns and social and demographic factors. Younger participants scored more highly for the Processed pattern and less highly educated participants had lower scores for the Fruit and vegetables pattern. These observations concur with other reports [33, 41, 42, 47], identifying younger and less educated groups as priorities for clinical and public health intervention. Whilst Black and Asian participants scored highly for the African/Caribbean pattern, these groups also had lower and therefore healthier scores for the Processed and Snacks dietary patterns which might redress the dietary balance. There is some conflict in the literature regarding dietary patterns amongst ethnic minority groups, likely a reflection of specific ethnic origin and different levels of acculturation. In agreement with the present findings, Northstone et al. showed that non-White women in the UK were less likely to score high for a ‘Confectionary’ dietary pattern [47], whilst Sommer et al. demonstrated that non-European pregnant women living in Norway were more likely to belong to unhealthier patterns [48]. Amongst a New Zealand population, higher scores for a ‘Junk’ pattern and lower scores for ‘Traditional’ pattern were found among Maori and Pacific Island ethnicities compared to European women [42]. Our data provide an additional example of distinctive dietary habits among ethnic minorities, illustrating the importance of understanding dietary patterns amongst ethnic groups when designing targeted health promotion interventions.

We also investigated the impact of maternal dietary patterns on pregnancy outcomes, finding that the Processed and African/Caribbean dietary patterns were associated with a significantly higher risk of developing GDM. Others have shown, as might be anticipated, that dietary patterns which consist of foods high in sugar and/or fat and high intakes of saturated fat and soft drinks are related to an increased risk of GDM [41, 49]. Glycemic load and a high intake of animal protein especially red meat has been reported to increase GDM risk [50, 51] which would concur with the increased risk observed for the African/Caribbean participants.

We previously reported that the UPBEAT intervention was effective in reducing GL and saturated fat intake [17] in the intervention group. In the present study, using an alternative assessment of dietary intake, we report a reduction in two unhealthy dietary patterns; Processed and Snacks. However, these dietary changes were insufficient to improve clinical outcomes including GDM. The outcomes of the UPBEAT and the LIMIT RCT in Australia have led to the suggestion that interventions in early pregnancy might be better focused on women known to at risk of adverse outcomes. Whilst, insulin resistance is higher in obese women at the beginning of pregnancy [52], only 25% develop GDM according to IADPSG criteria [17]. Development of an accurate early pregnancy risk assessment tool eg assessment of diet alongside other risk factors would enable targeted interventions, which might include diet, physical activity and/or pharmacological intervention for those individuals at greatest risk. Here, the identified dietary patterns in early pregnancy could be used as targets in those women who are likely to be motivated to change their diet than the general obese pregnant population.

Strengths of the study include the large sample size and this being the most intensive behavioral intervention to date to have focused on obese pregnant women. The participants were also amongst the highest priority groups for intervention because of their ethnic and social diversity, and associated higher risks of obesity and adverse pregnancy outcomes. To our knowledge, dietary pattern analysis has not been applied in an intervention in obese pregnant women; others have assessed changes in diet quality using scores and indices [14] but these were based on *a priori* criteria which might overlook components of the diet which may be open or resistant to change. The use of dietary pattern analysis is important for capturing some of the complexity of the diet while overcoming the limitations of single nutrient analysis [19].

This study provides evidence that specific dietary patterns in obese pregnant women are linked to gestational diabetes, however, there are some limitations, which must be considered. We acknowledge that the participants in the current study may have risk factors for the development of GDM other than dietary patterns, which have not been explored. Principally, in order to add power to our analysis, we have chosen to consider both intervention and control groups together at baseline and the insights derived from this study must be taken in this context. Furthermore, a significant proportion of women were not included in the dietary pattern analysis due to data incompatibility (pilot study participants) or incomplete data. Additional limitations include collection of dietary data using an FFQ which may be subject to bias [53], and factor analysis involves a number of arbitrary decisions including consolidation of food items into groups, the number of factors to extract, rotation method and naming of the factors [54]*.*

## Conclusions

We identified specific dietary patterns that were associated with an increased risk of GDM in obese pregnant women living in the UK, and have characterized sub-groups who were likely to follow these patterns. The UPBEAT intervention was effective in improving particular maternal dietary patterns, which could be targeted in future antenatal interventions which aim to lower risk of adverse outcomes in obese pregnant women.

## Additional files

Additional file 1: Table S1. UPBEAT targeted dietary advice. (DOCX 11 kb)
Additional file 2: Table S2. Food items considered for identification of dietary patterns. (DOCX 15 kb)
Additional file 3: Table S3. Factor loadings of items in the four dietary patterns identified. (DOCX 15 kb)

## Abbreviations

95% CI: 95% confidence intervals
ANCOVA: Analysis of covariance
BMI: Body mass index
CHO: Carbohydrate
EPIC: European Prospective Investigation into Cancer
FFQ: Food frequency questionnaire
GDM: Gestational diabetes
GI: Glycemic index
GL: Glycemic load
IADPSG: International Association of Diabetes and Pregnancy Study Groups
IOM: Institute of Medicine
LGA: Large for gestational age
SFA: Saturated fat
SGA: Small for gestational age
SMART: Specific, Measurable, Achievable, Relevant, Time Specific
UPBEAT: UK Pregnancies Better Eating and Activity Trial

## References

[1] World Health Organization. Obesity and overweight. Fact Sheet N°311. 2014. http://www.who.int/mediacentre/factsheets/fs311/en/. Accessed 3rd May 2016.

[2] Torloni MR, Betran AP, Horta BL, Nakamura MU, Atallah AN, Moron AF, Valente O (2009). Prepregnancy BMI and the risk of gestational diabetes: a systematic review of the literature with meta-analysis. Obes Rev.

[3] Nelson SM, Matthews P, Poston L (2010). Maternal metabolism and obesity: modifiable determinants of pregnancy outcome. Hum Reprod Update.

[4] Stuebe AM, Oken E, Gillman MW (2009). Associations of diet and physical activity during pregnancy with risk for excessive gestational weight gain. Am J Obstet Gynecol.

[5] Emmett PM, Jones LR, Golding J (2015). Pregnancy diet and associated outcomes in the Avon Longitudinal Study of Parents and Children. Nutr Rev.

[6] von Ruesten A, Brantsaeter AL, Haugen M, Meltzer HM, Mehlig K, Winkvist A, Lissner L (2014). Adherence of pregnant women to Nordic dietary guidelines in relation to postpartum weight retention: results from the Norwegian Mother and Child Cohort Study. BMC Public Health.

[7] Leventakou V, Roumeliotaki T, Martinez D, Barros H, Brantsaeter AL, Casas M, Charles MA, Cordier S, Eggesbo M, van Eijsden M (2014). Fish intake during pregnancy, fetal growth, and gestational length in 19 European birth cohort studies. Am J Clin Nutr.

[8] Shapiro AL, Kaar JL, Crume TL, Starling AP, Siega-Riz AM, Ringham BM, Glueck DH, Norris JM, Barbour LA, Friedman JJ, Dabelea D. Maternal diet quality in pregnancy and neonatal adiposity: The healthy start study. Int J Obes (Lond). 2016;40(7):1056–62. 10.1038/ijo.2016.79 PMC5356926 27133623

[9] Godfrey KM, Barker DJ (2001). Fetal programming and adult health. Public Health Nutr.

[10] Brion MJ, Ness AR, Rogers I, Emmett P, Cribb V, Davey Smith G, Lawlor DA (2010). Maternal macronutrient and energy intakes in pregnancy and offspring intake at 10 y: exploring parental comparisons and prenatal effects. Am J Clin Nutr.

[11] Heppe DH, Medina-Gomez C, Hofman A, Franco OH, Rivadeneira F, Jaddoe VW (2013). Maternal first-trimester diet and childhood bone mass: the Generation R Study. Am J Clin Nutr.

[12] Sausenthaler S, Koletzko S, Schaaf B, Lehmann I, Borte M, Herbarth O, von Berg A, Wichmann HE, Heinrich J (2007). Maternal diet during pregnancy in relation to eczema and allergic sensitization in the offspring at 2 y of age. Am J Clin Nutr.

[13] Okubo H, Crozier SR, Harvey NC, Godfrey KM, Inskip HM, Cooper C, Robinson SM. Maternal dietary glycemic index and glycemic load in early pregnancy are associated with offspring adiposity in childhood: the Southampton Women’s Survey. Am J Clin Nutr. 2014;100(2):676–83. 10.3945/ajcn.114.084905 24944056

[14] Dodd JM, Turnbull D, McPhee AJ, Deussen AR, Grivell RM, Yelland LN, Crowther CA, Wittert G, Owens JA, Robinson JS (2014). Antenatal lifestyle advice for women who are overweight or obese: LIMIT randomised trial. BMJ.

[15] Bain E, Crane M, Tieu J, Han S, Crowther CA, Middleton P (2015). Diet and exercise interventions for preventing gestational diabetes mellitus. Cochrane Database Syst Rev.

[16] Oteng-Ntim E, Varma R, Croker H, Poston L, Doyle P (2012). Lifestyle interventions for overweight and obese pregnant women to improve pregnancy outcome: systematic review and meta-analysis. BMC Med.

[17] Poston L, Bell R, Croker H, Flynn AC, Godfrey KM, Goff L, Hayes L, Khazaezadeh N, Nelson SM, Oteng-Ntim E, et al. Effect of a behavioural intervention in obese pregnant women (the UPBEAT study): a multicentre, randomised controlled trial. Lancet Diabetes Endocrinol. 2015;3(10):767–77. 10.1016/S2213-8587(15)00227-2 26165396

[18] Thangaratinam S, Rogozinska E, Jolly K, Glinkowski S, Roseboom T, Tomlinson JW, Kunz R, Mol BW, Coomarasamy A, Khan KS (2012). Effects of interventions in pregnancy on maternal weight and obstetric outcomes: meta-analysis of randomised evidence. BMJ.

[19] Hu FB (2002). Dietary pattern analysis: a new direction in nutritional epidemiology. Curr Opin Lipidol.

[20] Ocke MC (2013). Evaluation of methodologies for assessing the overall diet: dietary quality scores and dietary pattern analysis. Proc Nutr Soc.

[21] Briley AL, Barr S, Badger S, Bell R, Croker H, Godfrey KM, Holmes B, Kinnunen TI, Nelson SM, Oteng-Ntim E (2014). A complex intervention to improve pregnancy outcome in obese women; the UPBEAT randomised controlled trial. BMC Pregnancy Childbirth.

[22] Metzger BE, Gabbe SG, Persson B, Buchanan TA, Catalano PA, Damm P, Dyer AR, Leiva A, Hod M, Kitzmiler JL (2010). International association of diabetes and pregnancy study groups recommendations on the diagnosis and classification of hyperglycemia in pregnancy. Diabetes Care.

[23] Bingham SA, Welch AA, McTaggart A, Mulligan AA, Runswick SA, Luben R, Oakes S, Khaw KT, Wareham N, Day NE (2001). Nutritional methods in the European Prospective Investigation of Cancer in Norfolk. Public Health Nutr.

[24] Poston L, Briley AL, Barr S, Bell R, Croker H, Coxon K, Essex HN, Hunt C, Hayes L, Howard LM (2013). Developing a complex intervention for diet and activity behaviour change in obese pregnant women (the UPBEAT trial); assessment of behavioural change and process evaluation in a pilot randomised controlled trial. BMC Pregnancy Childbirth.

[25] Foster-Powell K, Holt SH, Brand-Miller JC (2002). International table of glycemic index and glycemic load values: 2002. Am J Clin Nutr.

[26] Aston LM, Jackson D, Monsheimer S, Whybrow S, Handjieva-Darlenska T, Kreutzer M, Kohl A, Papadaki A, Martinez JA, Kunova V (2010). Developing a methodology for assigning glycaemic index values to foods consumed across Europe. Obes Rev.

[27] Atkinson FS, Foster-Powell K, Brand-Miller JC (2008). International tables of glycemic index and glycemic load values: 2008. Diabetes Care.

[28] Food Standards Agency. Food Portion Sizes. Crawley H Ed. 3rd edition. London: The Stationery Office; 2002.

[29] Wrieden WL, Barton KL. Calculation and collation of typical portion sizes for adults aged 19–64 and older people aged 65 and over. Final Technical Report to the Food Standards Agency. 2006.

[30] Welch AA, Luben R, Khaw KT, Bingham SA (2005). The CAFE computer program for nutritional analysis of the EPIC-Norfolk food frequency questionnaire and identification of extreme nutrient values. J Hum Nutr Diet.

[31] Northstone K, Emmett PM (2008). A comparison of methods to assess changes in dietary patterns from pregnancy to 4 years post-partum obtained using principal components analysis. Br J Nutr.

[32] Flynn AC, Schneeberger C, Seed PT, Barr S, Poston L, Goff LM (2015). The effects of the UK Pregnancies Better Eating and Activity Trial Intervention on Dietary Patterns in obese pregnant women participating in a pilot randomized controlled trial. Nutr Metab Insights.

[33] Brantsaeter AL, Haugen M, Samuelsen SO, Torjusen H, Trogstad L, Alexander J, Magnus P, Meltzer HM (2009). A dietary pattern characterized by high intake of vegetables, fruits, and vegetable oils is associated with reduced risk of preeclampsia in nulliparous pregnant Norwegian women. J Nutr.

[34] Volgyi E, Carroll KN, Hare ME, Ringwald-Smith K, Piyathilake C, Yoo W, Tylavsky FA (2013). Dietary patterns in pregnancy and effects on nutrient intake in the Mid-South: the Conditions Affecting Neurocognitive Development and Learning in Early Childhood (CANDLE) study. Nutrients.

[35] Northstone K, Emmett PM, Rogers I (2008). Dietary patterns in pregnancy and associations with nutrient intakes. Br J Nutr.

[36] Rasmussen MA, Maslova E, Halldorsson TI, Olsen SF (2014). Characterization of dietary patterns in the Danish national birth cohort in relation to preterm birth. PLoS One.

[37] Laraia BA, Bodnar LM, Siega-Riz AM (2007). Pregravid body mass index is negatively associated with diet quality during pregnancy. Public Health Nutr.

[38] Moran LJ, Sui Z, Cramp CS, Dodd JM (2013). A decrease in diet quality occurs during pregnancy in overweight and obese women which is maintained post-partum. Int J Obes.

[39] Rifas-Shiman SL, Rich-Edwards JW, Kleinman KP, Oken E, Gillman MW (2009). Dietary quality during pregnancy varies by maternal characteristics in Project Viva: a US cohort. J Am Diet Assoc.

[40] Tsigga M, Filis V, Hatzopoulou K, Kotzamanidis C, Grammatikopoulou MG (2011). Healthy Eating Index during pregnancy according to pre-gravid and gravid weight status. Public Health Nutr.

[41] He JR, Yuan MY, Chen NN, Lu JH, Hu CY, Mai WB, Zhang RF, Pan YH, Qiu L, Wu YF, et al. Maternal dietary patterns and gestational diabetes mellitus: a large prospective cohort study in China. Br J Nutr. 2015;113(8):1292–300. 10.1017/S0007114515000707 25821944

[42] Thompson JM, Wall C, Becroft DM, Robinson E, Wild CJ, Mitchell EA (2010). Maternal dietary patterns in pregnancy and the association with small-for-gestational-age infants. Br J Nutr.

[43] Goff LM, Timbers L, Style H, Knight A. Dietary intake in Black British adults; an observational assessment of nutritional composition and the role of traditional foods in UK Caribbean and West African diets. Public Health Nutr. 2014;18(12):2191–201. 10.1017/S1368980014002584 PMC10271628 25412921

[44] Dodd JM, Cramp C, Sui Z, Yelland LN, Deussen AR, Grivell RM, Moran LJ, Crowther CA, Turnbull D, McPhee AJ (2014). The effects of antenatal dietary and lifestyle advice for women who are overweight or obese on maternal diet and physical activity: the LIMIT randomised trial. BMC Med.

[45] Greaves CJ, Sheppard KE, Abraham C, Hardeman W, Roden M, Evans PH, Schwarz P (2011). Systematic review of reviews of intervention components associated with increased effectiveness in dietary and physical activity interventions. BMC Public Health.

[46] Leung G, Stanner S (2011). Diets of minority ethnic groups in the UK: influence on chronic disease risk and implications for prevention. Nutr Bull.

[47] Northstone K, Emmett P, Rogers I (2008). Dietary patterns in pregnancy and associations with socio-demographic and lifestyle factors. Eur J Clin Nutr.

[48] Sommer C, Sletner L, Jenum AK, Morkrid K, Andersen LF, Birkeland KI, Mosdol A. Ethnic differences in maternal dietary patterns are largely explained by socio-economic score and integration score: a population-based study. Food Nutr Res. 2013;8:57. 10.3402/fnr.v57i0.21164 PMC3707086 23843779

[49] Schoenaker DA, Soedamah-Muthu SS, Callaway LK, Mishra GD. Pre-pregnancy dietary patterns and risk of gestational diabetes mellitus: results from an Australian population-based prospective cohort study. Diabetologia. 2015;58(12):2726–35. 10.1007/s00125-015-3742-1 26358582

[50] Zhang C, Liu S, Solomon CG, Hu FB (2006). Dietary fiber intake, dietary glycemic load, and the risk for gestational diabetes mellitus. Diabetes Care.

[51] Bao W, Bowers K, Tobias DK, Hu FB, Zhang C (2013). Prepregnancy dietary protein intake, major dietary protein sources, and the risk of gestational diabetes mellitus: a prospective cohort study. Diabetes Care.

[52] Catalano P, deMouzon SH (2015). Maternal obesity and metabolic risk to the offspring: why lifestyle interventions may have not achieved the desired outcomes. Int J Obes (Lond).

[53] Medical Research Council. Diet and Physical Activity Measurement Toolkit (DAPA). http://dapa-toolkit.mrc.ac.uk/. Accessed 15 May 2016.

[54] Martinez ME, Marshall JR, Sechrest L (1998). Invited commentary: Factor analysis and the search for objectivity. Am J Epidemiol.

