# Supplementary material for: Dietary patterns in obese pregnant women; influence of a behavioral intervention of diet and physical activity in the UPBEAT randomized controlled trial
Source: Int J Behav Nutr Phys Act. 2016 Nov 29;13:124. doi: 10.1186/s12966-016-0450-2 (PMC5126873; doi:10.1186/s12966-016-0450-2)
Supplement: Additional file 1: Table S1. — UPBEAT targeted dietary advice. (DOCX 11 kb) [file 12966_2016_450_MOESM1_ESM.docx]

**Additional file 1: Table S1 UPBEAT targeted dietary advice**

| **UPBEAT dietary changes** | **Instead of this…** | **Choose this…** |
| --- | --- | --- |
| 1. Soft drinks | Regular soft drinks | Water and sugar free drinks including herbal tea |
| 2. Sugar | Sugar | Choose fruit to sweeten foods or artificial sweeteners |
| 3. Bread | Any type of white or brown bread | Multigrain and granary breads |
| 4. Rice and potatoes | Rice, mashed potatoes, chips | Basmati rice, pasta or new potatoes |
| 5. Snacks | Chocolate, sweets, biscuits, cakes | Fresh fruit, low fat yogurts, cereal bars |
| 6. Breakfast cereals | Sugary cereals | Healthy cereals (UPBEAT recommended) or porridge |
| 7. Dairy products | Full fat dairy products | Lower fat dairy products |
| 8. Meat and meat products | Fatty meat and meat products including meat pies, burgers and sausages | Lean meat, chicken, fish, and beans and pulses |
