# Supplementary material for: Dietary patterns in obese pregnant women; influence of a behavioral intervention of diet and physical activity in the UPBEAT randomized controlled trial
Source: Int J Behav Nutr Phys Act. 2016 Nov 29;13:124. doi: 10.1186/s12966-016-0450-2 (PMC5126873; doi:10.1186/s12966-016-0450-2)
Supplement: Additional file 2: Table S2. — Food items considered for identification of dietary patterns. (DOCX 15 kb) [file 12966_2016_450_MOESM2_ESM.docx]

**Additional file 2: Table S2 Food items considered for identification of dietary patterns**

| Food item | Type | Examples |
| --- | --- | --- |
| Milk | Full fat | Whole milk, powdered, goats |
|  | Reduced fat | Semi,1%, skimmed, semi skimmed goats |
|  | Non dairy | Soya, rice, oat, almond |
|  | Evaporated/condensed | Evaporated, condensed |
|  |  |  |
| Cereal | Refined | Cornflakes, Cocopops, Rice krisipes, Crunchy nut, Frosties, Golden balls, Krave, Chocoflakes, Lucky charms, Curiously cinnamon, Special K, Sugar puffs, Cookie crisps, Golden nuggets, Honey loops, Nesquick, Lion bar cereal, Weetos, Clusters, Blueberry wheats, Frosted/honey cheerios, Frosted shreddies |
|  | Non-refined | All bran, Shredded wheat, Weetabix, Bran flakes, all types of porridge, Fruit n' fibre, Sultana bran, muesli, granola, Oatibix, Cheerios, Just right, Shreddies |
|  |  |  |
| Bread | White | White |
|  | Wholemeal | Wholemeal, brown, granary, seeded, best of both/50:50, oatmeal |
|  | Speciality | Bagel, pitta, baguette/French bread, chapati, brioche, croissant, tortilla/wrap, sourdough, rye |
|  | Cultural breads | African/Ghana/sugar bread/hard dough |
|  |  |  |
| Spread | Butter/full fat spread | Butter, margarine, Anchor, Lurpak, Clover |
|  | Reduced fat butter/spread | Low fat dairy spread/margarine, olive oil spreads, Lurpak lighter, Flora/Flora light/proactiv, Benecol, Clover light, Vitalite |
|  |  |  |
| Cheese | Full fat cheese | Hard, soft |
|  | Reduced fat cheese | Reduced fat, cottage, mozzarella, Philadelphia light, Dairylea light, Laughing cow |
|  |  |  |
| Drinks | Fruit juice | Fruit juice and juice drinks |
|  | Squash/fizzy drinks | Ribena, coke, lemonade |
|  | Sugar free squash/fizzy drinks | Ribena really light, diet coke |
|  | Tea/cofffee without added sugar |  |
|  | Tea/coffee with added sugar |  |
|  |  |  |
| Starch foods | White/brown/basmati rice |  |
|  | Pilau/fried/jollof rice |  |
|  | Pasta/noodles | Pasta/spaghetti (dried, fresh, stuffed), noodles (dried, fresh) |
|  | Old/new potatoes | Potatoes (‘new’/baby boiled, ‘old’ baked, boiled, roast or mashed) |
|  | Takeaway/oven chips | Takeaway chips, fries, potato products, oven chips |
|  | Cassava | Cassava, fu fu, gari, kenkey |
|  | Plantain | Plantain, yam |
|  |  |  |
| Vegetables | Salad | Salad leaves/lettuce, cucumber, tomatoes |
|  | Green | Broccoli, spring greens, kale, spinach, or other green vegetables |
|  | Root | Carrots, radishes, beetroot, turnip, parsnips |
|  | Lentils/peas/beans | Lentils, peas, beans |
|  |  |  |
| Fruit | Bananas |  |
|  | Citrus | Oranges, satsumas, mandarins, grapefruit |
|  | Fresh fruit | Apples, pears, grapes, fruit salad, berries |
|  | Tropical | Mango, pineapple, lychee, melon, figs, dates |
|  | Dried | Raisins, prunes |
|  |  |  |
| Snacks | Chocolate bar | Milk/plain/white eg Galaxy, Dairymilk, Twix, Mars |
|  | Cereal bar | Oat, fruit, nut, seed |
|  | Biscuits/cookies | Chocolate chip, digestive, custard cream |
|  | Cakes/pastries | Doughnuts, Danish pastries, sponge cake, muffin, gateau |
|  | Sweets | Jelly sweets, chewy sweets, boiled sweets |
|  | S. Asian sweets | Kheer, kulfi, burfi, jelabi, gulab jaman, falooda, ladoo |
|  | Crisps | Crisps |
|  | S. Asian fried snacks | Dosa, Pakoras, Bhaji, bhaturas, paratha. |
|  | Yoghurt | Yoghurt, fromage frais, lassi |
|  |  |  |
| Meat/fish | Red meat | Beef, pork, lamb, mutton, goat and other red meats |
|  | White meat | Chicken, turkey |
|  | Processed/ meat products | Beefburger, sausages, kebab, pork pie, sausage roll, pasty, coated/breaded chicken |
|  | Fish | Cod, haddock, plaice, sole, halibut, mackerel, kippers, salmon, sardines, herring |
|  | Fish products | Fish in batter/crumbs, fish fingers, fishcakes |
|  | Tofu | Tofu, soya meat, textured vegetable protein (TVP), Vegeburger |
