# Supplementary material for: Dietary patterns in obese pregnant women; influence of a behavioral intervention of diet and physical activity in the UPBEAT randomized controlled trial
Source: Int J Behav Nutr Phys Act. 2016 Nov 29;13:124. doi: 10.1186/s12966-016-0450-2 (PMC5126873; doi:10.1186/s12966-016-0450-2)
Supplement: Additional file 3: Table S3. — Factor loadings of items in the four dietary patterns identified. (DOCX 15 kb) [file 12966_2016_450_MOESM3_ESM.docx]

**Supplementary Table 3 Factor loadings of items in the four dietary patterns identified**

| **Food item** | **Fruit and veg** | **African/Caribbean** | **Processed** | **Snacks** |
| --- | --- | --- | --- | --- |
| Bananas | 0.383* | 0.053 | -0.110 | 0.113 |
| Red meat | 0.054 | 0.312* | 0.097 | 0.129 |
| Biscuits/cookies | 0.041 | 0.041 | 0.069 | 0.540* |
| Butter/full fat spread | -0.065 | -0.075 | 0.083 | 0.116 |
| Cakes/pastries | 0.053 | 0.034 | 0.058 | 0.555* |
| Cassava | -0.103 | 0.630* | -0.023 | 0.008 |
| Cereal bar | 0.026 | 0.212 | 0.023 | 0.244 |
| White meat | 0.014 | 0.364* | 0.166 | 0.145 |
| Chocolate | -0.119 | -0.044 | 0.248* | 0.555* |
| Citrus fruit | 0.635* | 0.021 | 0.019 | 0.060 |
| Crisps | -0.060 | -0.122 | 0.523* | 0.202 |
| Dried fruit | 0.305* | -0.053 | -0.150 | 0.069 |
| Evaporated/condensed milk | 0.023 | 0.106 | -0.079 | -0.048 |
| Fish products | 0.042 | -0.017 | 0.034 | 0.235 |
| Fresh fruit | 0.701* | -0.002 | -0.027 | 0.025 |
| Fruit juice | 0.244 | 0.175 | 0.021 | 0.172 |
| Full fat cheese | 0.018 | -0.166 | 0.053 | 0.335* |
| Full fat milk | -0.043 | 0.133 | 0.023 | 0.063 |
| Green vegetables | 0.541* | 0.062 | 0.281* | -0.101 |
| Pulses | 0.311* | 0.155 | -0.022 | 0.076 |
| Non dairy milk | 0.042 | -0.049 | -0.113 | 0.102 |
| Non refined breakfast cereals | 0.165 | -0.004 | -0.179 | 0.136 |
| Potatoes | 0.231 | -0.067 | 0.566* | 0.067 |
| Pasta/noodles | 0.102 | 0.098 | 0.032 | 0.196 |
| Pilau/fried/jollof rice | -0.035 | 0.467* | 0.034 | -0.031 |
| Plantain | 0.098 | 0.522* | -0.049 | -0.062 |
| Processed/meat products | 0.046 | 0.042 | 0.476* | 0.188 |
| Reduced fat butter/spread | 0.011 | -0.078 | 0.016 | 0.156 |
| Reduced fat cheese | 0.021 | -0.035 | -0.026 | -0.029 |
| Reduced fat milk | 0.060 | -0.135 | 0.005 | 0.063 |
| Refined breakfast cereals | -0.076 | -0.060 | 0.040 | 0.031 |
| Root vegetables | 0.499* | 0.078 | 0.322* | -0.098 |
| Salad vegetables | 0.441* | 0.003 | 0.206 | -0.056 |
| S. Asian fried snacks | 0.070 | 0.109 | 0.072 | 0.044 |
| S. Asian sweets | 0.050 | 0.109 | 0.003 | 0.173 |
| Speciality breads | 0.112 | -0.052 | -0.099 | 0.036 |
| Squash/fizzy drinks | -0.011 | -0.034 | 0.427* | 0.187 |
| Sugar free drinks | -0.015 | -0.096 | 0.386* | 0.133 |
| Sugar honey | 0.044 | 0.013 | -0.092 | 0.108 |
| Sweets | 0.005 | -0.027 | 0.137 | 0.315* |
| Takeaway/oven chips | 0.132 | 0.008 | 0.679* | -0.001 |
| Tea/coffee with sugar | 0.030 | 0.040 | -0.018 | 0.093 |
| Tea/coffee without sugar | 0.065 | -0.059 | -0.016 | 0.086 |
| Tofu | 0.049 | -0.014 | -0.071 | 0.070 |
| Tropical fruit | 0.494* | 0.072 | 0.167 | -0.046 |
| White bread | -0.059 | -0.002 | 0.124 | 0.159 |
| White/brown/basmati rice | 0.100 | 0.656* | -0.066 | -0.003 |
| Fish | 0.106 | 0.573* | -0.038 | 0.006 |
| Wholemeal bread | 0.069 | -0.011 | -0.003 | 0.116 |
| Yoghurt | 0.298* | -0.030 | -0.060 | 0.166 |

*Loadings ≥ ±.25
